# Beyond empathy: a qualitative exploration of arts and humanities in pre-professional (baccalaureate) health education

**DOI:** 10.1007/s10459-020-09964-z

**Published:** 2020-02-25

**Authors:** Marcela Costa, Emilia Kangasjarvi, Andrea Charise

**Affiliations:** 1 Federal University of Pernambuco, Recife, Brazil; 2 SCOPE: The Health Humanities Learning Lab, University of Toronto Scarborough, Scarborough, Canada; 3 Centre for Faculty Development, Faculty of Medicine, University of Toronto at St. Michael’s Hospital, Toronto, Canada; 4 Interdisciplinary Centre for Health & Society (ICHS), University of Toronto Scarborough, 1265 Military Trail, c/o Highland Hall Rm. 220, Toronto, ON M1C 1A4 Canada; 5 Department of Psychiatry, Faculty of Medicine, University of Toronto, Toronto, Canada

**Keywords:** Baccalaureate, Capability approach, Epistemological multicompetence, Health humanities, Health professions education (HPE), Arts and humanities, Medical humanities, Pre-professional, Empathy, Undergraduate medical education

## Abstract

For nearly four decades, researchers have explored the integration of arts and humanities content into health professions education (HPE). However, enduring controversies regarding the purpose, efficacy, and implementation of humanities initiatives suggest that the *timing* and *context* of trainees’ exposure to such content is a key, but seldom considered, factor. To better understand the affordances of introducing humanities-based health curriculum prior to the HPE admissions gateway, we conducted a qualitative instrumental case study with participants from Canada’s first Health Humanities baccalaureate program. Fully anonymized transcripts from semi-structured interviews (n = 11) and focus groups (n = 14) underwent an open-coding procedure for thematic narrative analysis to reveal three major temporal domains of described experience (i.e., *prior to, during*, and *following* their participation in a 12-week semester-long “Introduction to Health Humanities” course). Our findings demonstrate that perceptions of arts- and humanities content in health education are generated well in advance of HPE admission. Among other findings, we define a new concept—epistemological multicompetence—to describe participants’ emergent capability to toggle between (and advocate for the role of) multiple disciplines, arts and humanities particularly, in health-related teaching and learning at the pre-professional level. Improved coordination of baccalaureate and HPE curricula may therefore enhance the development of capabilities associated with arts and humanities, including: epistemological multicompetence, aesthetic sensibility, and other sought-after qualities in HPE candidates. In conclusion, attending to the pre-professional admissions gateway presents a new, capabilities-driven approach to enhancing both the implementation and critical understanding of arts and humanities’ purpose, role, and effects across the “life course” of health professions education.

## Introduction

For nearly four decades, researchers have investigated the integration of arts and humanities content into undergraduate, graduate, post-graduate, and continuing medical and health professions education (HPE) more broadly. However, the implementation of such methods and materials in these educational contexts remains contentious. To briefly summarize this research: proponents of “medical humanities” have argued that by encouraging attention to less overtly clinical aspects of healthcare, humanities-engaged clinical education helps build social and relational skills, often by fostering empathy and moral development; compassion; self-reflection; interpersonal skills; perspective-taking; openness to otherness; critical reflection; and tolerance for ambiguity (Kleinman 1988; Charon 2001; Kumagai et al. 2007; Kumagai 2008, 2012; Deloney and Graham 2003; Kumagai and Lypson 2009; Kumagai and Wear 2014; Bates et al. 2014; Bleakley 2015; Kinsella and Binosti 2016; Ofri 2017; Mangione et al. 2018). Commonly associated with “soft” skills relevant to clinical practice, arts and humanities disciplines (which may involve history, philosophy, literary studies, and fine arts, to name a few; for brevity, we will refer to “humanities” throughout this article) can be generally described as prioritizing aesthetics, social experiences, and interpretive methods over the quantitative modes of investigation more typical to empirically-oriented STEM subjects (such as biology, chemistry, and physics) (Becher and Trowler 2001; Marra and Palmer 2008).

Despite these promising—and frequently idealized—claims, there is still no settled consensus regarding the purpose, role, efficacy, or optimal implementation of arts- and humanities-based content in the HPE context (Williams 2019; McFarland et al. 2018). To rehearse a well-known example: one controversial literature review concluded that “evidence on the positive long-term impacts of integrating humanities into undergraduate medical education is sparse” (Ousager and Johannessen 2010, p. 998), in large part because of the difficulty measuring the realization of teaching outcomes in medical education, and the elusiveness of measurable or quantitatively-reported learning outcomes for assessing the arts and humanities’ “impact” on learners. In response to such conclusions, critics countered that “the value of the humanities in educating new physicians can be defended by demonstrating the need for more complex approaches to knowledge than complete dependence on empirical evidence” (Belling 2010 p. 938). “Rather than beg off the need to measure these teaching efforts,” Charon (2010) concludes, “teachers in the humanities can embrace the demanding task of delineating how medicine changes when fortified by narrative competence and humanities-derived skills.” (p. 935) And so the debate continues, as the studies, commentaries, and publications proliferate: how can practitioners better articulate how humanities-based clinical education mitigates critical issues at the individual and institutional level of health care—wicked problems that include physician burnout prevention and management, professionalism, and supporting patient-centered health care? (Gillis 2008; Shapiro et al. 2009; Doukas et al. 2012, 2015; Jones et al. 2017; Gillies 2018; Mangione et al. 2018).

Here, we argue, lies a critical problem. An enormous volume of literature exists on the purported utility of the humanities, the need to integrate humanities-based content into HPE, and challenges and failures in realizing its purported goals in this setting (McFarland et al. 2018). However, as we contend here, an ongoing fixation on *what humanities*-*based educational initiatives are good for* in the HPE context essentially overlooks a foundational issue. To be blunt: more than 40 years of unresolved controversy regarding the role of humanities-based content in HPE suggests that there must be something amiss with the way this field has framed the matter of integrating such material into health professions education.

This study offers an alternative interpretation and reframing of the ongoing “debate” concerning the role of humanities in health professions education. Conspicuously lacking in discussions to date is any consideration of the *timing* and *context* of trainees’ exposure to humanities content: whether the introduction of such curriculum in, say, undergraduate medical education is optimal or, indeed, effective. Consider the well-known issues of time-pressured undergraduate medical curricula, the contested status of reflective “portfolios” (be they for-credit, optional, or mandatory; Ng et al. 2015), and the generally diminished status of humanities-focused content in medical education. Under such constraints, we must ask: is medical school really the moment to inset humanities methods and materials into a health professional’s “life course”? Is it really possible to implement meaningful, impactful, and intellectually-sound humanities training at the health professions level? Added to this is the unfortunate reality that throughout North America, “arts and humanities are progressively being marginalized from the academic curricula” of K-12 and postsecondary education and that, consequently, “including the humanities of any type into medical schools, although convenient, may be too little too late” (Morera Serna, p. 12). Without prior exposure to a set of disciplinary conventions that significantly diverges from conventional “pre-med” preparation, it seems unlikely that new, unfamiliar, humanities-based skills and knowledge can be successfully integrated, taught, or valued—at the undergraduate level or beyond.

We propose that the enduring problems of introducing humanities in medical and health professions education are, in large part, the consequence of an implementation error: that is, even when introduced at the undergraduate level, humanities education likely comes too late to offer much transformative impact or benefit. Counterintuitive as it may seem, when considering how humanities content may be better positioned within HPE, it may be more strategic to redirect our focus beyond the HPE admissions gateway. Doukas et al. (2015) for example, call for a more focused attention to medical school’s admission process that encompasses “pre-professional readiness” of ethics and humanities, which might encourage “premedical learners to appreciate exposure to philosophy, anthropology, sociology, and psychology as valuable preparation toward medical education, including the groundwork of critical thinking skill building” (p. 739). Simultaneously, recent research has begun to explore how interdisciplinary training at the baccalaureate level impacts pre-professional students’ understanding of health (Schwartz et al. 2009; Crawford et al. 2015; Baker et al. 2017; Barron 2017; Berry and Lamb 2017; Charise 2017; Hirshfield et al. 2019; Jones et al. 2019; Wald et al. 2019).

An opportunity therefore exists in the fact that little attention has been paid to the *optimal timing* of implementing humanities-based content in the larger context of health education. Given the rise of pre-professional Health Humanities programs across North America (Lamb and Berry 2017), our goal is to explore whether *baccalaureate* health education offers new, actionable insights into how to best implement arts- and humanities-based content relevant to HPE and clinical practice. We argue that introducing health-related humanities content at the pre-professional, baccalaureate level may optimize the introduction of such concepts, when the potential for impacting conventional disciplinary health knowledge and training is greater (in contrast to the later setting of medical or health professions education). Importantly, this study is not especially concerned with whether or not such education has the effect of producing “better” doctors, health professionals, or improved clinical care. Instead, by exploring the impact of Health Humanities at the baccalaureate level, our goal is to (1) reset the terms of a stalled debate concerning the role of humanities in HPE, and (2) provoke a new, evidence-driven conversation regarding the temporal relationship of HPE and the baccalaureate training that forms its critical foundation.

This study develops scholarly evidence regarding the impact of teaching and learning Health Humanities at the pre-professional baccalaureate level, by exploring the reported effects of such curriculum on a more junior and general population than is typically reported in the HPE literature. This instrumental qualitative case study asks: How do students in Canada’s first baccalaureate Health Humanities program describe their experiences of a humanities-based approach to health education? Our aim was to explore pre-HPE students’ perceptions of their experience in order to better understand the potential affordances of introducing humanities-based health curriculum at this moment in the curricular timeline. To ensure clarity of purpose and application of our findings (Crawford et al. 2010), throughout this study we will employ the term “Health Humanities” to distinguish between pre-professional (i.e., baccalaureate) health education curricula—the focus of this study—versus the primarily clinical emphases of “medical” humanities more typical to HPE research.

## Methods

The University of Toronto Scarborough (UTSC)’s baccalaureate Health Humanities curriculum launched in 2014 (for program information, including courses and learning objectives, see www.scopelab.ca). Located in the northeastern part of Toronto, a major Canadian city described as one of the most multicultural in the world, UTSC is also home to higher-than-average numbers of students identifying as part of racialized, recent immigrant, or low-income communities. At the time of participant recruitment, students from any disciplinary background or program were eligible to enroll in the semester-long course “Introduction to Health Humanities.” As per registrar requirements, however, preference was given to students with declared majors in UTSC’s “Health Studies” program.

Approval was granted by the University of Toronto’s Office of Research Ethics (#32777) to conduct an instrumental qualitative case study (Stake 1995). An instrumental case study was determined to be an appropriate method because of its ability to use a particular instance of a phenomenon as a way to provide insight into the larger phenomenon it represents (Stake 1995). The site was chosen because of its position as the nation’s first (and currently only) curriculum of its kind at the baccalaureate level.

### Participant recruitment

Between March and May 2016, undergraduate students who had recently completed “Introduction to Health Humanities” (two cohorts within the preceding 3–12 months) were recruited using the following techniques: (1) targeted group email to current UTSC Health Humanities Listserv members (approximately 70 undergraduate student members at time of recruitment); (2) in-person announcements made in other Health Humanities classes during recruitment period; (3) dissemination of recruitment materials through relevant student association mailing lists and social media pages (e.g., Facebook); (4) limited paper postering at the study site, including relevant faculty, departmental, and student association offices. Inclusion criteria was having completed the introductory course in Health Humanities at the study site, and the ability to understand and converse in English.

20 students were recruited online or following in-class announcements. An additional 5 students were recruited by snowball sampling. Informed consent was obtained from all individual participants included in the study.

### Pre-existing researcher–participant relationships

The principal investigator (AC) was previously the instructor of all researched participants (i.e., undergraduate students or recent program alumni). To mitigate this power relation, at all stages of the study the PI was blocked from direct contact with potential or confirmed participants and remained blind to participants’ identifying data.

### Data collection

To maximize convenience and participation, participants elected to undertake either a one-on-one semi-structured interview (n = 11) or participate in a focus group (n = 14) between April and June 2016.

Interviews and focus groups used the same semi-structured question framework instrument, which was developed in light of informal discussions between co-authors as well as broader issues raised in scholarly literature regarding interdisciplinary health education (Klein 1990, 2005). Questions aimed to draw out participants’ reflections on their experience, such as: *What made you decide to take Health Humanities? How would you convince a friend to take Health Humanities? What would you say to someone who was reluctant or unsure about taking Health Humanities,* and *Looking back at your experience, what does taking Health Humanities make you capable of?*

Average duration for semi-structured interviews was 28 min (range: 17–53 min) and 63 min for focus groups (range: 26–100 min). Demographic information collected included participants’ gender, year of birth, major/minor program, and year of study (see Table 1). Approximately 11 months post-data collection, 18/25 participants engaged in a member-checking focus group to verify research findings.

**Table 1:** Summary of participant demographics (N = 25)

Gender	
Female	21
Male	4
Age (y)	
Range	19–24
Mean	22.2
Completed years of Baccalaureate study	
1–2	3
3–4	17
5+	4
Declared Baccalaureate Major or field of study (note: students may have multiple majors)	
Health Studies	15
Medicine	5
Psychology	4
Mental Health	3
Human Biology	2
Health Policy	2
Cellular and Molecular Biology	1
Neuroscience	1
Public Health	1
Sociology	1
Women’s Studies	1
English Literature	1
Diaspora Studies	1
Linguistics	1
Declared Baccalaureate Minor (note: students may have multiple minors or none)	
Psychology	3
Anthropology	3
English Literature	2
Biology	2
Economics	1
French	1

### Analysis

Data analysis employed fully anonymized transcripts of interviews and focus group discussions, in addition to moderator notes taken during and immediately following the sessions. We followed a standard qualitative content analysis approach (Patton 2002; Reissman 2008) beginning with the extraction of key descriptive content from direct-answer questions.

To enhance credibility of findings, the author with no participant contact (AC) initiated an open-coding procedure for thematic narrative analysis to identify potential codes using an initial set of interview transcripts. The primary codebook was generated by authors independently coding all interview transcripts to identify emerging themes. Differences were resolved by consensus.

To further inform the coding structure, we then independently conducted more directed coding techniques (see Saldaña 2009), including: *structural* (a content-based or conceptual phrase representing a topic of inquiry or segment of data), *versus* (to identify meaningful binary terms or conflicts), *affective* (which investigates subjective qualities of human experience—such as emotions and values—by directly acknowledging and naming those experiences), and *descriptive* coding (a word or short phrase summarizing the topic of segment of qualitative data). Using these codes, two authors then independently determined main thematic response categories using the full suite of transcripts in MS Excel.

Data triangulation occurred through cross-verification of transcripts, field notes, and member-checking. Recruitment ceased when no new themes or findings were evident in the data; data saturation occurred in close alignment with the theoretical saturation point proposed in the research ethics documents and relevant scholarly literature, which was twenty (Mason 2010; O’Reilly and Parker 2012). Total participant recruitment for this study was 25.

## Results

The results of our case study revealed three major temporal domains of anticipated and realized experience. Participant responses clustered into descriptions of experience *prior to, during*, and *following* their participation in a 12-week semester-long baccalaureate Introduction to Health Humanities course. These temporal domains gave rise to the three main categories described below: “Approaching Health Humanities,” “Encountering Health Humanities,” and “After Health Humanities”. For an abbreviated summary of categories and themes described below, see Table 2.

**Table 2:** Summary of qualitative results organized by temporal domain and theme

Temporal domain	Themes
1. Approaching Health Humanities: Confronting Forced Choices	a. Charting Disciplinary Real Estate
	b. The Humanities Brand: Draws (*Novelty, Previous positive relationship, Perceived applicability to health, Curricular necessity*) and Deterrents (*Unfamiliarity, Diminished disciplinary prestige, Fear of failure, Gendered content)*
	c. Enticements to Interdisciplinarity (*Disciplinary estrangement* vs *Epistemological alienation*)
2. Encountering Health Humanities: “Real Life Adult” Ambivalence	a. “You just sit there”: Passivity, Compliance, and Conventional Health Education
	b. Redrawing Sites of Disciplinary Knowledge Authority
	c. Humanities as Practical Training
	d. “It’s a double-edged sword”: Interdisciplinarity and Creative Community Building
3. “You get this humanity touching you”: After Health Humanities	a. Cultivating Epistemological Multicompetence
	b. Managing Complexity and Affective Entanglement
	c. Developing Aesthetic Capability
	d. “A newfound respect for the arts”

Findings described below elaborate this temporal clustering of themes with descriptive notes and illustrative participant quotations; quotations include non-identifying participant number, age in years, announced major and minor fields of study (as appropriate).

### Approaching health humanities: confronting forced choices

This domain comprised three major themes—*Charting Disciplinary Real Estate, The Humanities Brand: Draws and Deterrents,* and *Enticements to Interdisciplinarity*—that described participants’ perspectives and anticipated experiences prior to enrolling in Health Humanities.

Although addressing different issues, each theme reflects a repeated structural pattern: namely, participants characterizing their experiences of baccalaureate education as a series of two-alternative forced choices. *Before* the course especially, the pre-professional baccalaureate educational experience was perceived as an accumulation of forced choices (arts vs science, creativity vs protocol, essays vs tests, interest in a subject vs “good grades”, and so on). Furthermore, navigating these forced choices throughout participants’ baccalaureate education generally reflected a depreciation of the value or purpose of the arts and humanities.

#### a. Charting disciplinary real estate

Participants often described baccalaureate education as a spatially and temporally limited resource (“*I was always passionate about theatre… but I’ve never had a space for that in university*”), and felt obligated to ration time dedicated to health education by deprioritizing arts and humanities:

It’s been coming down from generations upon generations, like, you can either become an engineer or a lawyer, or a doctor or a teacher, right? You have to choose your path. You can’t have the arts and the sciences together. (I3, age 21, Health Studies and Human Biology)

#### b. The humanities brand: draws and deterrents

The term “brand” functions in two ways here: first, “positively” (or neutrally) as something that identifies or differentiates (i.e., a “draw”), and second, “negatively,” as a stigmatized mark (i.e., a “deterrent”). While specific draws and deterrents differed significantly between participants, the following were typically articulated as draws to the humanities brand: Novelty, Previous Positive Relationship, Perceived Applicability to Health, Curricular Necessity.

(i) Novelty

Well, first of all, like, it was a course that I never encountered before. When I read just the course title itself, I was like “Introduction to Health Humanities, okay, what is that?”, so I was intrigued by that. So, I was like, let’s attend the first lecture and let’s see how this goes. (I3, age 21, Health Studies and Human Biology)

(ii) Previous Positive Relationship to Humanities

I’ve always been a creative person, but I’ve never had the opportunity to really make that flare up in school. (I7, age 22, Medicine)

(iii) Perceived Applicability to Health

One of the things I’ve been really talking to them [my friends] about was… the fact that it’s a course where the arts and the sciences are coming together. And, when I tell them that, they’re like “Are you kidding me? Are you serious? This course exists?” (I3, age 21, Health Studies and Human Biology)

(iv) Curricular Necessity

I finished all my major requirements and I needed an elective… instead of doing something from another discipline [I thought] I might as well do something that is from health. (FG1-4, age 23, Health Policy with double minor in Psychology and Anthropology)

By contrast, notable deterrents included: Unfamiliarity, Diminished Disciplinary Prestige, Fear of Failure, Gendered (and specifically feminized) content. Note that some draws and deterrents are versions or the inverse of each other; for example, for some participants “Novelty” (a draw noted above) connoted “Unfamiliarity,” a deterrent, which might turn away students who prefer to stay within their known strengths and/or disciplinary training.

(i) Unfamiliarity

It’s such a new field that you don’t know what to expect. Oh, it’s “Health Humanities” [physically gestures with air quotes], but what does that actually mean? Because those two words side by side, it’s new, we don’t know what that means. (I6, age 23, Health Studies and Women’s Studies)

However, the keyword “health” had a familiarizing effect for some participants **(**“*the title would just make a lot of students do it, because it says health. I feel like a lot of students took it just because it said health on there*”).

(ii) Diminished Disciplinary Prestige

Participants described perceptions of the arts and humanities as lesser in status and desirability than other disciplinary subjects. Several described being “*turned off by the word humanities*”:

Oh, man, the arts are just not taken seriously… they’re considered “soft”, or not needed, or just something for fun and not science, which is something that I myself thought, even though I’ve always loved the arts. (I7, age 22, Medicine)

(iii) Fear of Failure

Several participants articulated a pre-emptive assessment anxiety associated with humanities, particularly forms of assessment that involved writing and more complex forms of communication and knowledge synthesis:

Health humanities was one of the first courses in university where I had to write an essay. So, that was a stressful process for me… I think one of the other things that someone might be reluctant about is… if they think creativity or artistic expression is required of the course… I don’t like to be forced to do things. (I9, age 22, Neuroscience and Human Biology)

(iv) Gendered Content

Some participants shared their sense of arts and humanities as being gendered, and specifically feminized (“*it’s an automatic girl thing*”), with transparently negative associations:

I think there’s also something going on with… not just Health Humanities courses but with humanities, health studies. They say that it’s more feminist type of courses or programs…. there’s this stereotype associated with these kind of courses, so … that might discourage somebody. (FG1-4, age 23, Health Policy with double minor in Psychology and Anthropology)

(v) Perceived Workload

Perceived workload (either higher or lower than conventional health education) was more ambivalently perceived as either a draw or deterrent.

#### c. Enticements to Interdisciplinarity (*Disciplinary estrangement* vs *Epistemological alienation*)

Two distinct, yet associated, predispositions impacted participants’ interest in Health Humanities: what we defined as “disciplinary estrangement” and “epistemological alienation.” *Disciplinary estrangement* describes some participants’ sense of estrangement from conventional (i.e., biomedical) disciplinary values of health education. For example:

I was really looking for other discussions of health, so not biological discussions, not epidemiological discussions. (I9, age 22, Neuroscience and Human Biology)

I remember asking, “is there a point where you can combine [art and health]?”… There is. So I’m not just in my head a crazy person who’s trying to come up with ideas. (I3, age 21, Health Studies and Human Biology)

By contrast, *epistemological alienation* described participants’ aversion toward disciplinary conventions, assessment methods, and values of the humanities (what might be more generally described as participants’ hesitancy to be involved with the humanities “brand” described above).

I had never done really well in the English courses in high school, and I dreaded it. I just wanted to run away in the opposite direction, you know, from any English courses… I’m not good in humanities, these types of things. I don’t wanna take it. (I4, age 20, Cellular and Molecular Biology and Public Health)

### Encountering health humanities: “real life adult” ambivalence

This domain comprised four major themes—*Passivity, Compliance, and Conventional Health Education; Redrawing Sites of Disciplinary Knowledge Authority; Humanities as Practical Training; Interdisciplinarity and Creative Community Building*—that described participants’ recollections of encountering Health Humanities in the classroom setting. In summary, participants’ recalled experience of their time spent within a baccalaureate Health Humanities learning environment were characterized by their recognition of significant differences—often interpreted as strongly positive or favourable distinctions—from conventional approaches to health education. Participants tended to view their baccalaureate encounter with Health Humanities as a practical form of acquired knowledge, while simultaneously expressing several risks and rewards to the field’s perceived creative freedom.

#### a. “You just sit there”: passivity, compliance, and conventional health education

In contrast to their experience of a Health Humanities course, participants regularly described conventional baccalaureate health education—both its teaching and learning contexts—as disenfranchising or passive:

[In] a normal health studies course… you go to class every single day, you don’t necessarily have to give your opinion, you just listen to the professor and watch the slides or the powerpoint or whatever. Then you study off that and then for your final and your mid-term and you get your grade and that’s it. (I8, age 24, Health Studies with double minor in Biology and Psychology)

It’s always easier for a professor to get a book, build some slides, slideshow, present that to their students: “so this is cancer, this is breast cancer, these are the genes that are altered in most cases, this is the treatment, this is the prognosis, so many people die, so many people survive, goodbye, see you next week.” Um, it is easy. It is easy to teach and it is easy to learn this way, but it is not necessarily interesting. (I1, age 23, Medicine)

Some participants drew strong parallels between baccalaureate health education and the reality (imagined or experienced) of health professions work:

A lot of people [in Health Humanities] were like “I don’t like that the assignments make you, basically, think and personally reflect.” And, I know that a lot of people, especially people going into medicine or in the higher sciences are just really anxious doing that, probably just because they don’t have much experience doing it in a classroom setting. But I think that’s probably why people don’t take [Health Humanities], because it’s not an easy A at all. […] You have to actually spend time thinking about what you’re reading and how it relates to the world around you, so I think that’s why people are reluctant to take it. (I11, age 21, Health Studies with double minor in English Literature and Anthropology)

#### b. Redrawing sites of disciplinary knowledge authority

Participants associated their baccalaureate exposure to Health Humanities with new sites of learning, suggesting a change in prior perceptions of limited “disciplinary real estate” described in the first temporal domain above. Participants described Health Humanities as introducing new relationships to evidence and sources of disciplinary knowledge authority, particularly with respect to the humanities’ contributions to health knowledge:

I would say that [Health Humanities is] very English-y, and it’s different, because [of] this essay writing… it’s lovely, and it feels like you’re back almost in English class. But, you’re learning health unintentionally, like, you don’t think you’re learning it… It’s not like “Yeah, I’m learning science here”, so I think that’s what it’s unique about it. And, I’ve taken intro to public health, biomedical ethics, they all tend to bring you back to science and science and science, but this… takes science concepts and it brings it back to an English perspective. (I4, age 20, Cellular and Molecular Biology and Public Health)

#### c. Humanities as practical training

This category describes participants’ understanding of specific purposes for the humanities relevant to their understanding of health (“*Works of art… can serve a purpose. Like, we’re not just making art because it’s pretty or because it’s entertaining [but] because it makes people feel better, makes people feel less isolated or, like, makes people’s illness experiences better. That’s something that I never would’ve learned without taking a course like this*”). References to the “instrumental”, “practical”, or “applied” nature of humanities included the value of stories and storytelling practices both within and outside of the health-related context:

I compared this course to a real-life adult course. You know how in high school and first year nobody actually teaches you how to be an adult. You don’t know how to file your taxes or you don’t know how to apply for a credit card, or even talk to like a bank manager to get what you need, right, loans and everything. I had to learn this all by myself… this course kind of gives you that real-life understanding of what it’s like to be in the healthcare field. (FG1-5, age 23, Psychology and Health Studies)

#### “It’s a double-edged sword”: interdisciplinarity and creative community building

Participants consistently described Health Humanities teaching and learning as “*accessible*” and epistemologically inclusive (i.e., of students’ multiple disciplinary backgrounds). Different disciplinary backgrounds were further acknowledged as a source of diversity in the classroom. Stories were seen as a form of inclusion even though storytelling was an unfamiliar aspect of conventional baccalaureate health education:

When I was taking this course, I talked a lot to my family and my friends… we were able to have conversations that related to it. I never explained to them exactly what we did in class but it’s relatable to everyone. Everyone I talked to, they were all interested in it because of the stories they told. We all appreciate hearing a good story or watching a good movie. So, like, it wasn’t mechanical. People I talked to were interested even though it wasn’t their field. (I6, age 23, Health Studies and Women’s Studies)

I really liked the fact that the things were—there was room for creativity in a way that wasn’t being graded so much. There was just participation so you didn’t feel as much pressure. So, for a person that really is a perfectionist, it’s really hard to put myself out there and be creative, so it was a challenge for me. (I9, age 22, Neuroscience and Human Biology)

Stories were generally seen as a vehicle of inclusion, however, one notable tension emerged. Positive affirmations of creativity associated with humanities-based health education were countered by some participants’ description of anxieties or frustrations that accompany a more creative, “*unprescribed*” (I3) approach:

It’s… a double-edged sword. I mean, writing the papers, you need to think about it, I need to make these connections, but I don’t actually understand or don’t feel it or see it through. That, I found a little difficult. But I also loved writing the papers. (I5, age 24, Mental Health and Health Studies)

I don’t want to sound rude saying this, [but] I was really frustrated with some other students in the class. Like, it’s so good that everyone can speak their mind and stuff, but I often found that, like … the entire lecture is spent listening to people’s opinions… it just, like, frustrated me a bit, at times… I kind of found myself wishing there were almost, like, two streams of [Health Humanities]. (FG2-2, age 21, Health Studies with double minor in English Literature and Anthropology)

This was the most extreme example of such dissatisfaction, but it was met with nods of agreement in this focus group and similar remarks surfaced in other data collection sessions. For some participants, the improved inclusivity of humanities-based approaches to baccalaureate health education (i.e., searching for others “like me” in terms of tolerance for arts- and humanities-based content) often involved rejecting or dismissing peers either as credible sources of authority, expertise, or story-makers.

### “You get this humanity touching you”: after health humanities

This final temporal domain comprised four major themes–*Cultivating Epistemological Multicompetence; Managing Complexity and Affective Entanglement; Developing Aesthetic Capability* “*A newfound respect for the arts*”—which described participants’ reflections on the longer-term outcomes of experiencing humanities-based content as an approach to health education.

#### a. Cultivating epistemological multicompetence

Participants’ responses often linked the ability to toggle between disciplines, or to understand the role of multiple disciplines (especially arts and humanities) in generating more complex knowledge of health and illness. We defined this emergent practice as “epistemological multicompetence,” to refer to the willingness and/or ability to apprehend and work alongside different disciplines. Such capacity further suggested an intellectual component to cultivating empathy in interdisciplinary setting, as opposed to “putting oneself in another’s shoes” (Berger 1987), as empathy has long been described in the health education literature:

I have a lot of friends, because I’m doing a major in human bio as well… for them it’s always like, science, science, bio[logy], genetics, microbio, blah blah blah. And I always say that it’s very cool, it’s awesome that you’re doing this, but, if you take a Health Humanities course, it’ll provide you with that alternate perspective of health. You need a foundation in biology in your curriculum. But I feel like [Health Humanities] provides a more practical experience as to how you can deal with certain situations. A lot of my friends are interested in health care, they wanna pursue a career in the health care system. So I’m always like, “Hey guys, I really feel like you should take this course, because I feel like you can apply it, and it would provide you with a foundation to grow yourself within the health care field in a different way.”

A lot of my friends have done that, and they really enjoyed it. […] I tell them two things: one, the fact that [Health Humanities] is gonna provide you with a foundation, so, it is a health course, and it’s a really different perspective on health care […]. Secondly, it’s a combination of the arts and sciences. You’ve never experienced that in your major—why not take it now? (I3, age 21, Health Studies and Human Biology)

I would say “Man, just try to be a little more open, because you see that everyone needs to be in contact with humanities, or with the part of the knowledge of the world.” […] Because, uh, even though we are, as I said, more science [students], we are in contact with human beings and we have to be able to express ourselves and to understand what they are expressing. So once you get this humanity touching you, it definitely makes things easier.” (I10, age 21, Medicine)

In contrast to epistemological alienation (described in temporal domain 1c “Enticements to Interdisciplinarity”), epistemological multicompetence describes participants’ direct confrontation with, and subsequent navigation of, different disciplinary knowledges: here, the experience of determining the arts and humanities’s productive relationship to health knowledge. What distinguishes this particular theme is the way such an encounter catalyzes an *articulated advocacy* for such learning approaches *by learners themselves*: explicitly with respect to the purpose and value of arts- and humanities knowledge within health education.

#### b. Managing complexity and affective entanglement

This theme describes a trend in participants’ shift in tolerance for complexity: from the tidy and restrictive demarcations of disciplinary real estate and affective “distance” prior to the course, towards a recognition of the complexities of health and illness experience:

What I took away [from the course] was that health, in a stereotypical way is, “you’re sick, we need to fix it.” From a humanities aspect, it’s like,… you need to sort of conjure [health], keep it soft, and safe. So, what the humanities taught me was, if you need to fix something, address the problem—but also know that you can’t just, it’s not a one [time] fix. You need other factors and disciplines and emotions to fix things as well. (I5, age 24, Mental Health and Health Studies)

Notably, despite recognizing the often challenging affective entanglements created by the complexity of health knowledge and practice, participants did not appear to indicate negative outcomes such as overwhelming emotions, moral and/or empathic distress (Adamson et al. 2018), or hopelessness. This ability was related directly to anticipated practices as future health care professionals:

Your illness is a story, how you restore your health is a story… I aspire to be a doctor, and seeing the lack of connection between doctors and their patients, the fact that doctors and medical professionals, health professionals, aren’t able to connect with the stories of patients, is what really bothered me. That’s something I picked up [from this course], and that’s something I hope to integrate in my life if I become a doctor, hopefully. You know, to better understand my patients and not see them as number 395 on the records. (I4, age 20, Cellular and Molecular Biology and Public Health)

#### c. Developing aesthetic capability

This theme describes participants’ discussion of their own development of sense-attention, imagination, and creative thought as a generative, valuable, and often pleasurable activity. Rather than regarding the heightening of aesthetic sensitivity as antithetical to health-related knowledge and practice, participants described how such capabilities helped generate meaningful, educational, practicable knowledge:

I think I can read facial expressions better, I can notice nuances in skin color better, which is something that we call “close noticing.” I think close noticing is something that is of vital, terrific importance to health and medicine. And, to me, that is something that the Health Humanities made me capable of. To really explore the words, the tone of voice that the patient used, the expression, the look in their eyes. I think I’m extremely sensitive to that now. (I7, age 22, Medicine)

#### d. “A newfound respect for the arts”

Participants reported a greater sense of the value of arts and humanities more generally (“*I got back into [reading] after a long time*”). In addition to describing its applicability to health, for several participants the arts and humanities are newly valued in their own right:

The importance of the arts: I feel like that is one of the most important things I learned. Because art is always pushed away on the side, like, it’s all about the science … I feel like I have a newfound respect for the arts through Health Humanities, […] I thought that that was something very valuable that I’ve learned overall. (I3, age 21, Health Studies and Human Biology)

I think that the basic utility of the arts is to socialize and share the emotional aspects of someone’s story and make all kinds of different life stories familiar to the world at large. And it’s always more impactful when you see something being portrayed in an art form. (I7, age 22, Medicine)

## Discussion

Our findings indicate that baccalaureate health education serves, and is often openly understood, as a preparatory space for integrating the knowledge, skills, and values of arts- and humanities-based health education at the professional level. The three major temporal domains that organized our data—namely, participants’ recollections of experience *prior to, during*, and *following* their enrolment in a semester-long baccalaureate “Introduction to Health Humanities” course—help clarify how knowledge of and attitudes toward arts- and humanities content in health education are generated in advance of the HPE admissions gateway.

Prior to taking the course (temporal domain 1: “Approaching Health Humanities: Confronting Forced Choices”), we can see how disapproving trends regarding humanities implementation in HPE (such as not seeing the value or utility of humanities in health care education, or experiencing “humanities teaching as pointless, boring, worthless, or just plain stupid” (Shapiro et al. 2009, p. 192; Wear and Zarconi 2006) are seeded at the baccalaureate level. As subtheme 1c, “Enticements to Interdisciplinarity” indicates, the tension between “disciplinary estrangement” (which described disenchantment with biomedical disciplinary values of conventional health education) and “epistemological alienation” (which described aversion toward disciplinary conventions of the humanities) marked a critical moment in baccalaureate education: one where attitudes or opinions concerning the values of arts and humanities may be shaped, and, significantly, remain malleable as a means of demonstrating their relevance to health education.

During the course (temporal domain 2: “Encountering Health Humanities: ‘Real Life Adult’ Ambivalence”), some critique of conventional health education is apparent (as in subtheme 2a, “Passivity, Compliance, and Conventional Health Education”) as participants recall their exposure to the use-value of the humanities (subtheme 2c, “Humanities as Practical Training”) and creative potential of interdisciplinary health education (subtheme 2d, “Interdisciplinarity and Creative Community Building”). The “risk” of devaluing the arts and humanities remains, as the “double-edged sword” (subtheme 2d) of engaging in interpretive, more open-ended arts- and humanities-based entails an acquaintance with new disciplinary conventions (subtheme 2b, “Redrawing Sites of Disciplinary Knowledge Authority”). Despite such ambivalence, however, our findings indicate that these potentially negative trends—which are regularly cited as implementation issues facing humanities-based content in HPE (Wear and Zarconi 2006; Garden 2007; Tsevat et al. 2015; Wald et al. 2019)—are not necessarily indelible at this earlier moment in the longer curricular timeline.

The third and final temporal domain (“‘You get this humanity touching you’: After Health Humanities”) involved participants’ reflections on their perceived capabilities as a result of taking a humanities-based health course, often with reference to anticipated careers and/or continuing education in the health professions. Significantly, following completion of the baccalaureate course, participants’ assessment of the humanities’ value in health education coalesced around positive indicators and supportive arguments for the role and purpose of such content. More unexpected was the subtheme finding 3d (“A newfound respect for the arts”), which demonstrated that exposure to Health Humanities at the baccalaureate level had the effect of elevating participants’ admiration for and, in some cases, spontaneous participation in arts and humanities-based activities elsewhere in their lives. This finding is a vital counter-response to concerns that arts and humanities are effectively instrumentalized (Macnaughton 2000; Belling 2010; Blease 2016; Chiavaroli 2017) or reduced in intellectual density when deployed in the context of health education, especially at the professional level. Our study suggests instead that exposure to high-quality humanities-based health education at the baccalaureate level—by appropriately credentialed faculty with recognized qualifications in both health-related and arts/humanities fields, in curricular offerings deliberately designed in response to relevant educational scholarship, pedagogically-sound learning objectives, critical debates and evidence-driven issues in the field—may in fact equip students to serve as advocates: not only of the value of arts and humanities in the health context, but in de-instrumentalized terms relevant to broader societal and cultural engagements of the arts and humanities as well.

This study contributes to the development of empirical data regarding the role of arts and humanities within the longer “life course” of health education, beginning at the pre-professional baccalaureate level. In addition to presenting meaningful data for researchers, educators, and post-secondary program administrators for improving the robustness of baccalaureate Health Humanities programs, our findings include important lessons for practitioners interested in improving the uptake of humanities content in HPE. While we want to be clear that there has been much valuable work done to establish and enhance the success of humanities initiatives in the post-baccalaureate HPE context, there is enough continuing debate in this field to justify our sense that the *timing* and *context* of trainees’ exposure to the humanities must be more comprehensively considered as a contributing factor to this sense of research and implementation impasse. Tsevat and colleagues (2015) have described how humanities programming in undergraduate medical education can make learning experiences “unsettling, disturbing, and even dangerous” (p. 1462) due to topics that require reflection on sometimes intimate or even intrusive personal stories; citing a study done by Shapiro et al. (2009), the authors assert “humanities can pose a threat to students by forcing them to examine their own vulnerability and uncertainty.” (p. 193). Without significant attention to the pre-professional readiness of HPE candidates to engage in humanities-based training, we risk accelerating, perhaps even solidifying, negative nodes of ambivalence that are evidently already seeded in conventional health education at the baccalaureate level (as mapped by our results, especially in temporal domains 1 and 2).

By contrast, if humanities content is rigorously introduced at the baccalaureate level—with dedicated attention to the matter of why and how humanities matter to HPE concerns and clinical practice—there exists an opportunity to reshape the disciplinary “real estate” that typically structures health education across the curricular life course. Wald et al. (2019) have described the enduring “dualism of medicine”, and acknowledge that although technical skills are required in health care, significant value must be placed on capacities that prepare future health care professionals for the messy “human complexity” and the “gray, uncertainty” of medicine (p. 495). Implementing Health Humanities in an intellectually and ethically rigorous curriculum at baccalaureate level (Berry and Lamb 2017) may enhance the “homeliness” of humanities for HPE candidates where they still have the space—both intellectually and in a curricular sense—to experience the methods and materials of the humanities and its value in a supported, lower-stakes context. Once again, it appears, timing is everything: and the best time to integrate humanities content into HPE may be well in advance of the admissions gateway.

Our findings provide further insight into why baccalaureate engagement with Health Humanities is so crucial to realizing its potential in the HPE context. In the first theme of temporal domain 3, we coin a new concept—epistemological multicompetence—to describe participants’ emergent ability to toggle between disciplines, that is, to understand the role of multiple disciplines (and particularly the arts and humanities) in generating more nuanced knowledge of health and illness. We borrow the term “multicompetence” from the scholarship of language acquisition, which refers to an individual’s ability to attain, and dynamically integrate knowledge of, more than one language in contrast to the monolingual, “native” speaker (Cook 2008). Importantly, the multicompetence perspective understands one person’s ability to work with multiple languages as a single connected system with multi-directional influences, rather than as a simple “sum” of separately acquired, isolated languages. Working from this established concept and, sensing a productive metaphor, our findings point to the existence of what we term *epistemological* multicompetence in the interdisciplinary health education context: a term we use to describe one’s ability to “toggle” between disciplinary knowledges and epistemological values (Charise 2017), here, in the service of understanding the role of multiple disciplines (i.e., arts and humanities) in generating more complex knowledge of health and illness. Much like language acquisition, developing epistemological multicompetence is *possible* at any stage of the curricular timeline. However, much like language (where acquisition and subsequent fluency is most likely in the early years of life), it appears to hold true that beyond the baccalaureate “window” there exist additional, likely significant, challenges to the uptake and development of epistemological multicompetence later in the curricular timeline. What meaningfully distinguishes epistemological multicompetence from other, potentially cognate descriptions that appear in the literature (such as “holistic”, “biopsychosocial”, “compassionate”, or “reflective” knowledge and practices, for example), is the way such an encounter catalyzes an *articulated advocacy* for arts- and humanities-based learning approaches *by learners themselves*: explicitly with respect to communicating the “epistemological value” (Chiavaroli 2017, p. 19) of the arts and humanities. This is a distinct, and hitherto untheorized, concept in the educational literature, not least of all because it leads with the benefits *relevant to the arts and humanities* instead of clinical competencies or outcomes more typical to the medical and health professional education literature.

Epistemological multicompetence further involves individual willingness to apprehend and work alongside different disciplines: a capacity that suggests a strongly intellectual component to cultivating empathy in interdisciplinary setting. As opposed to the well-worn notion of “putting oneself in another’s shoes,” as empathy has long been described in the health literature (Berger 1987), epistemological multicompetence engages directly with skeptical assessments of “empathy training” as the *raison d’être* of Health Humanities (Garden 2007; Baruch 2017). Given common—yet increasingly contested—references to “empathy” as the purpose and outcome of arts- and humanities in the context of health education (see, for example, Garden 2007; Charise 2017; Baruch 2017), we propose epistemological multicompetence as one approach to evolving the critical trajectory and theoretical robustness of these debates. In their article “Beyond Sparking Joy: A Call for a Critical Medical Humanities” (published in *Academic Medicine*, 2019), Zoe Adams and Anna Reisman write that “Medical humanities programs must shrug off the security blanket that language like ‘enrichment’ and ‘empathy’ provides”. As valuable an outcome as empathy enhancement may be, positing it as the *inevitable* outcome of exposure to humanities—or allowing it to stand as a uncritical, vaguely feelings-based moral imperative for such work—does little to advance our understanding of how, and why, arts and humanities might have beneficial effects in the context of conventional health education: or how best to implement them so that their potential is realized in this context.

“Epistemological multicompetence” usefully complicates, while building on, notions of “empathy” by introducing an intellectual facet (epistemology) to the conceptually unclear, indeed ethically problematic, “feeling” that often undergirds discussions of empathy (Garden 2007; Charise 2017; Baruch 2017; Adams and Reisman 2019). It also provides a concrete instance of the ways epistemology and empathy might equally deserve consideration in the context of humanities-based health education at the professional level. “Conceptualising empathy as a clinical skill is likely to lead to training in communication and professionalism skills. By contrast, an emphasis on the epistemological value of empathy (in the context of a humanities programme in medicine), or a particular ‘way of knowing’, would focus on the crucial understanding of patient circumstances and perspectives that could inform appropriate clinical practice” (Chiavaroli 2017). As described in temporal domain 3a (theme: “Cultivating Epistemological Multicompetence”) in describing the ability to work with and alongside (or with knowledge of) other disciplinary epistemologies, epistemological multicompetence is the opposite of epistemological alienation (described in temporal domain 1c “Enticements to Interdisciplinarity”) precisely because it confronts the reality of different disciplinary knowledges: here, the work of finding the arts and humanities useful or important as these disciplines relate to health knowledge. This reveals an association of disciplinary diversity with principles of social inclusion, since participants described as “accessible” the areas of the course that were richest in epistemological inclusivity (for example, stories of health and illness).

Epistemological multicompetence also lays the groundwork for an enhanced kind of individual agency that effectively counteracts the passivity and compliance typical to conventional health education (theme 2a), as some participants described their own emergent interests in mentoring and modeling such epistemological multicompetence for their peers (recall 3a, “*So I’m always like,* “*Hey guys, I really feel like you should take this course, because I feel like you can apply it, and it would provide you with a foundation to grow yourself within the health care field in a different way*”). As a result, epistemological multicompetence in interdisciplinary health education in fact requires a very real form of interdisciplinary empathy especially relevant to the Health Humanities field—a far more convincing argument than the typical, yet theoretically and practically suspect, claims that exposure to humanities is a method for enhancing “compassion” in the health setting (as if ingesting a few doses of poetry could solve systemic issues of burnout, professional misconduct, and lapses in patient-centered care). As valuable an outcome as empathy enhancement may be, our study identifies other fundamental reasons that arts and humanities might benefit conventional health education using a fresh and, importantly, evidence-driven rationale. If, as McFarland, Markovina, and Gibbs (2018) have argued, the debate concerning humanities’ purpose is ongoing and that “one of the main issues is how to introduce it into medical education” (p. 3), then developing epistemological multicompetence as a core capability prior to the HPE admissions gateway may help address questions of implementation, purpose, and value added in the HPE context. We propose, therefore, that the deliberate development of epistemological multicompetence should be a widespread objective—that is, a core capability—encouraged across the curricular spectrum of arts- and humanities-based health education (i.e., from baccalaureate to postgraduate and continuing HPE).

Notice our use of the term “capability” here. We employ it deliberately as a way of pointing to the potential advantages offered by a “capabilities approach” to humanities-based interdisciplinary health education, and—like others who have employed the language of capability in medical education (Mylopoulos and Woods 2017)—as an alternative to the contested language of “competencies” in medical education (despite its apparent manifestation in our discussion of epistemological multicompetence above). A social and economic theoretical framework primarily associated with economist-philosopher Amartya Sen and philosopher Martha Nussbaum, a capabilities approach emphasizes what individuals are able to do (i.e., what one is capable of) as an approach to more inclusively defining human wellbeing. Nussbaum’s articulation (2006, 2011) of ten central human capabilities that should be supported in all democratic contexts—namely, life; bodily health; bodily integrity; senses, imagination and thought; emotions; practical reason; affiliation; other species; play; and control over one’s environment—has some compelling relevance to our discussion here. Most immediately, Nussbaum’s citation of “senses, imagination and thought” informs theme 3c (“Developing Aesthetic Capability”), which refers to participants’ discussion of their own development of sense-attention, imagination, and creative thought as a generative, valuable, and often pleasurable activity, which is further directly relevant to their enhanced health-related knowledge.

We argue that Nussbaum’s ten central human capabilities provide an alternative conceptual framework in which to assert the significance of developing aesthetic sensibilities in health context (in contrast to, say, ongoing appeals to the arts as a means to “enhance empathy” or “compassion”). Nussbaum’s framework also highlights another problematic deployment of humanities education at the HPE level, which regularly requires trainees to develop and perform competencies in aesthetically-centered domains, when students are unlikely to have had much formal encouragement to develop such skills prior to the HPE gateway. Our findings show that the role and significance of the arts and humanities in health are aligned with the realization of central human capabilities; therefore, advocating for the role of the arts and humanities with regard to this principle, rather than the struggling, and not altogether convincing terms of “empathy training” or “compassion,” fundamentally reframes the arts as a practical, secular, and democratic goal for HPE and the baccalaureate programs it draws from. Clearly, what is needed is far more open lines of communication, program building, and capabilities development between arts and humanities educators, baccalaureate health programs, and HPE programs. Only with these crucial stakeholders at the table can we imagine enhancing the uptake of arts and humanities methods and materials in the health setting; to realize its potential outcomes, such work must begin well in advance of the HPE admissions gateway.

There are, of course, limitations to this study. Findings are based on self-reported retrospective data, and participants were self-selecting (rather than randomly selected to participate), and consequently likely more positively predisposed. However, given the investigator’s role as an educator alongside research responsibilities, arguing for students’ mandatory participation in an educational study at the baccalaureate level would be an ethically contentious approach to recruitment given institutional concerns about participant vulnerability. In terms of other limitations, it is known that focus group outcomes may be influenced by dominant member(s) (Morgan 1988); while facilitator training was implemented to manage such scenarios, group dynamics will inevitably influence the outcome of qualitatively collected data. More generally, an observer or Hawthorne effect (what Paradis and Sutkin have termed “participant reactivity”, 2017) may influence individual as well as group reactions to interview questions; our efforts to minimize such effects took the form of ensuring that the interview and focus group facilitator was a peer, and that student-participants understood that their identifying information would remain unidentifiable to the PI. Future research might elaborate and extend our findings by implementing a more experimental and randomized study design; tracking participants longitudinally over the course of baccalaureate (and where possible, HPE curriculum) to assess the longevity and/or changes to these findings over the curricular lifecourse; and exploring the existence of other moments of enhanced receptivity to humanities-based content and education (for example, in the wake of a personal critical incident or ethically-challenging clinical experience).

## Conclusion

Teaching and learning Health Humanities at the baccalaureate level is a critical moment in professional development, because attitudes towards arts and humanities are more malleable and subject to external conditions that may enhance—or diminish—perceptions of their value. As a result, baccalaureate training in arts and humanities presents a critical moment for future HPE candidates to integrate, and even advocate for, the role of arts and humanities in their trajectory as future health care professionals. At a time when the humanities are regularly devalued in health education (Morera 2018; Williams 2019) and are facing increasing pressure—or even elimination—in K-12 and postsecondary education, our findings demonstrate that key challenges with implementing humanities-based initiatives in HPE are already seeded in the baccalaureate context. Improving the coordination of arts and humanities educators, baccalaureate health programs, and HPE programs may therefore enhance the development of capabilities associated with arts and humanities as they pertain to HPE objectives present and future. An early and ongoing capabilities-driven approach—including the development of what we call “epistemological multicompetence”—may significantly enhance pre-professional readiness for sought-after, humanities-associated qualities in HPE candidates, while also introducing new opportunities for critical nuance regarding the purpose, role, and effects of integrating arts and humanities across the long curricular life course of health education.
